# The public health impact of COVID-19 variants of concern on the effectiveness of contact tracing in Vermont, United States

**DOI:** 10.1038/s41598-024-68634-x

**Published:** 2024-08-01

**Authors:** François M. Castonguay, Brian F. Borah, Seonghye Jeon, Gabriel Rainisch, Patsy Kelso, Bishwa B. Adhikari, Daniel J. Daltry, Leah S. Fischer, Bradford Greening, Emily B. Kahn, Gloria J. Kang, Martin I. Meltzer

**Affiliations:** 1 National Center for Emerging and Zoonotic Infectious Diseases, Division of Preparedness and Emerging Infections, Health Economics and Modeling Unit, Centers for Disease Control and Prevention, U.S. Department of Health and Human Services, Atlanta, GA USA; 2 Modeling Support Team, Contact Tracing and Innovation Section (CTIS), State Local Tribal and Territorial (STLT) Task Force, CDC COVID-19 Response; Centers for Disease Control and Prevention, Department of Health and Human Services, Atlanta, GA USA; 3 Department of Health Management, Evaluation and Policy, University of Montreal School of Public Health, and Centre for Public Health Research – CReSP, 7101 Avenue du Parc, 3e étage, Montréal, QC H3N 1X9 Canada; 4 Vermont Department of Health, Burlington, USA; 5 Epidemic Intelligence Service, U.S. Department of Health and Human Services, Centers for Disease Control and Prevention, Atlanta, GA USA

**Keywords:** Case investigation, Contact tracing, Cases averted, COVID-19, Modeling, Epidemiology, Preventive medicine, Health policy

## Abstract

Case investigation and contact tracing (CICT) are public health measures that aim to break the chain of pathogen transmission. Changes in viral characteristics of COVID-19 variants have likely affected the effectiveness of CICT programs. We estimated and compared the cases averted in Vermont when the original COVID-19 strain circulated (Nov. 25, 2020–Jan. 19, 2021) with two periods when the Delta strain dominated (Aug. 1–Sept. 25, 2021, and Sept. 26–Nov. 20, 2021). When the original strain circulated, we estimated that CICT prevented 7180 cases (55% reduction in disease burden), compared to 1437 (15% reduction) and 9970 cases (40% reduction) when the Delta strain circulated. Despite the Delta variant being more infectious and having a shorter latency period, CICT remained an effective tool to slow spread of COVID-19; while these viral characteristics did diminish CICT effectiveness, non-viral characteristics had a much greater impact on CICT effectiveness.

## Introduction

Case investigation and contact tracing (CICT) are public health tools that aim to interrupt the spread of infectious pathogens by identifying and contacting cases and contacts, and recommending that they isolate and quarantine, respectively, to prevent onward transmission^1^. Several modeling studies estimated that CICT had varying degrees of success in controlling COVID-19 transmission across US jurisdictions^2–4^ in the earlier phases of the pandemic, when the original strain of SARS-CoV-2 (the virus that causes COVID-19) primarily dominated circulation. However, additional studies have shown that the evolving epidemiological parameters of COVID-19 potentially affected our ability to control the pandemic^5,6^. There were two major virologic differences between the original strain of SARS-CoV-2 (hereafter for simplicity, strain, or variant, of COVID-19) and the Delta strain that circulated in the fall of 2021, that might have affected COVID-19 epidemiology. First, the time interval between infection and becoming infectious (*i.e.,* the latent period) is shorter by one day^7,8^ for the Delta variant. Second, the average number of infections caused by an infected individual in a population that has no immunity or protection against infection (*i.e.,* the basic reproduction number, *R*_*0*_) is estimated to be 5.0 for the Delta strain compared to 2.5 for the original strain^9,10^. The reduced latent period of the Delta strain implies that CICT must reach contacts earlier in order to have the same impact as when the original strain circulated. Alternatively, the higher transmissibility of the Delta variant could potentially increase CICT’s ability to prevent COVID-19 cases (hereafter, CICT effectiveness) since each averted case prevents more new infections than with the original strain, but it can simultaneously impose an increased caseload burden on CICT staff. The degree to which these factors offset one another is unclear.

Using data from Vermont’s CICT program, we compare estimates of CICT’s impact to avert COVID-19 cases in Vermont during a period of original SARS-CoV-2 strain predominance and two periods of Delta variant predominance. Using these estimates, we explore whether observed differences over time in CICT impact are explained by changes associated with the Delta variant in the virologic characteristics including *R*_*0*_ and latent period duration, or whether other factors such as population vaccination levels, public compliance with isolation and quarantine recommendations, and speed of CICT contact notification mostly influence CICT impact.

At the request of the Vermont Department of Health, in this paper we examine how strain-related variations in the transmissibility of COVID-19 impacted the effectiveness of CICT. This assessment will aid public health practitioners to better understand the impact of CICT with evolving COVID-19 variants and help develop CICT program strategies that can address future outbreaks of respiratory diseases like COVID-19.

## Material and methods

### Overview

**Figure 1:**
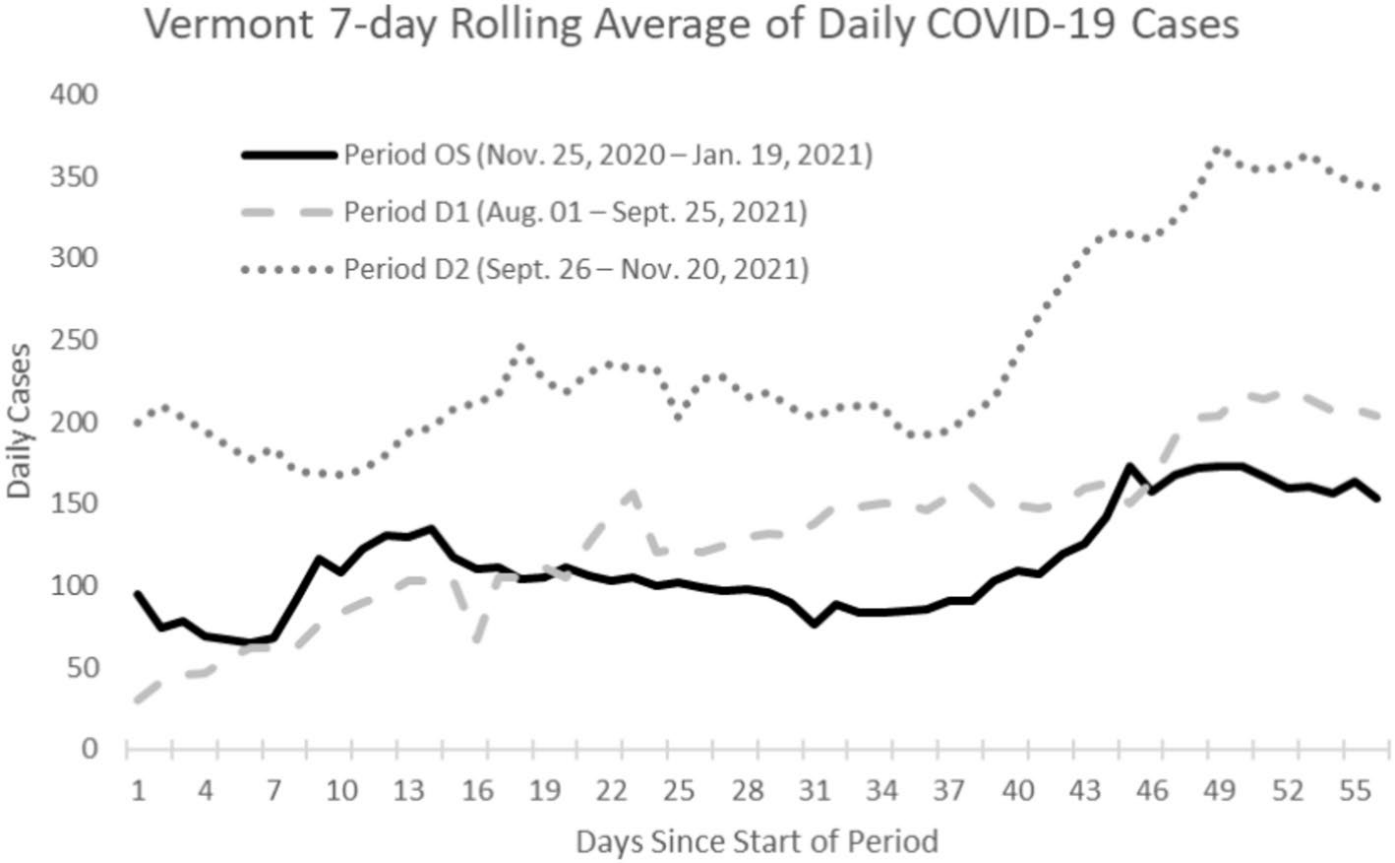
Oberved seven-day rolling average of daily COVID-19 cases in Vermont, for the three study periods (Period OS, Period D1, and Period D2), 2020–2021. Note that polymerase chain reaction (PCR) test—considered the gold standard for the detection of COVID-19^29^—represented 86.30% of all positive lab tests in Period OS, compared to 88.68% and 91.63% for Period D1 and Period D2, respectively (Table S1). Note that these three study periods represented time periods with similar effective reproduction number^30^. Some data outside the study periods were used to plot the beginning of the 7-day rolling average.

We used the U.S. Centers for Disease Control and Prevention (CDC)’s COVIDTracer Advanced modeling tool^11^ in combination with reported case data and CICT performance data from Vermont to estimate cases averted by CICT activities. This tool has previously been used to estimate the impact of CICT^2–4,12^ and other public health interventions^13^. COVIDTracer Advanced uses an epidemiological model to illustrate the spread of COVID-19 and the effects of community interventions. The tool allowed us to attribute reductions in transmission to either CICT or a combination of all other non-pharmaceutical interventions (NPIs) (additional details below). We focused on three distinct, 8-week time periods. The first was when the original COVID-19 strain circulated (Period OS: Nov. 25, 2020–Jan. 19, 2021), and the two subsequent periods were when the Delta strain dominated (*i.e.*, the Delta variant represented 99% or more of sampled strains in Vermont; Period D1: Aug. 01–Sept. 25, 2021; Period D2: Sept. 26–Nov. 20, 2021) (Fig. 1)^14^. The use of described 8-week periods (Fig. 1) balances the need for sufficient time to pass to allow for the compounding effects of CICT on future generations, while limiting the influence of other interventions over longer periods^2–4^. The selection of these specific periods aimed to balance the need to have substantially different variant characteristics (here, comparing the original strain to the Delta variant), while maintaining comparable case counts to minimize potential bias due to resource limitations in CICT that are not accounted for in our model. This activity was reviewed by CDC and was conducted consistent with applicable federal law and CDC policy (see *e.g.*, 45 C.F.R. part 46, 21 C.F.R. part 56; 42 U.S.C. §241(d); 5 U.S.C. §552a; 44 U.S.C. §3501 et seq.).

### Model

The CDC’s COVIDTracer Advanced tool is a deterministic Susceptible-Exposed-Infectious-Recovered (SEIR) epidemiological model that estimates the impact of interventions in a user-defined population (see Technical Appendix in online Supplementary Information for a detailed description of the model). The tool allows users to enter COVID-19 case count data and CICT performance metrics, and lets users set COVID-19 epidemiologic parameters (*e.g.,* latent period, duration of infections, number of persons infected in a totally susceptible population) (Table 1). Population levels of existing immunity, either due to previous infection and/or vaccination is accounted for by reducing the initial number of persons susceptible to infection (Table 1). The relatively short time step of each period analyzed means that population levels of immunity are unlikely to notably change during each period (note that the variation in vaccination coverage from the beginning to the end of Period D1 represents a 2.1 percentage points increase—roughly 13,000 people vaccinated—and a 3.1 percentage points increase for Period D2—roughly 19,500 people vaccinated; these increases are well within the range of the sensitivity analyses we performed for vaccination coverage in Fig. S1). COVIDTracer Advanced models all infections, including infectious asymptomatic cases. We assumed that 40% of all infections were asymptomatic and that asymptomatic cases infected 75% as many new cases as did symptomatic cases (see Table 1 for more details and for references). We assumed that once fully vaccinated, vaccine effectiveness against the Delta variant was 88%^15^. We also assumed that vaccination provided protection for exactly 180 days^16^ (Table 1)—see Supplementary Information for further details. We defined fully vaccinated as either having received two doses of the monovalent mRNA BNT162b2 (Pfizer-BioNTech, Comirnaty) or monovalent mRNA mRNA-1273 (Moderna, Spikevax) COVID-19 vaccine, or one dose of the single-dose adenovirus vector-based Ad26.COV.S (Janssen [Johnson & Johnson]) COVID-19 vaccine^17^.

**Table 1:** Comparison during the different time periods in Vermont, by dominating SARS-CoV-2 variant transmission, 2020–2021.

Dominant strain	Original strain	Delta variant	Delta variant
Study period (Dates covered)	Period OS* (Nov. 25, 2020–Jan. 19, 2021)	Period D1 (Aug. 01–Sept. 25, 2021)	Period D2 (Sept. 26–Nov. 20, 2021)
**Assumed epidemiological characteristics**			
Latent period duration^†^	3 days	2 days	2 days
Basic reproduction number^‡^	*R*_*0*_ = 2.5	*R*_*0*_ = 5	*R*_*0*_ = 5
% cases asymptomatic^§^	40%	40%	40%
Infectiousness of asymptomatic cases^¶^	75%	75%	75%
% persons vaccinated^#^	< 1%	66%	68%
Vaccine effectiveness**	–	88%	88%
Immunity duration^††^	180 days	180 days	180 days
**Reported number of days from infection to isolation or quarantine, median**^‡‡^	7 days	9 days	7 days

*The CICT program metrics during the period of original strain were provided by the CDC’s Epidemiology and Laboratory Capacity for Prevention and Control of Emerging Infectious Diseases program.

^**†**^Per the literature, we used a 3-day latent period during the period of the original strain^25^ and a 2-day latent period during the Delta wave^7,8^.

^‡^Per the literature, we used a basic reproduction number of 2.5 for the original strain^26,27^, while we used a value of 5.0 during the Delta wave^9,10^.

^§^Asymptomatic cases. Patients can be infected, and become infectious, without being symptomatic. They can and likely do contribute to onward transmission of the pathogen. Source: CDC and the Office of the Assistant Secretary for Preparedness and Response (ASPR/HHS): COVID-19 Pandemic Planning Scenarios: Updated September 10, 2020. Archived at: https://www.cdc.gov/coronavirus/2019-ncov/hcp/planning-scenarios.html#five-scenarios (accessed 26 October 2022).

^¶^Infectiousness of asymptomatic cases relative to symptomatic cases. Source: Same as for Percentage asymptomatic cases (above).

^#^Based on CDC COVID-19 vaccination data^28^—these data represent overall coverage among all ages. For Period OS (Nov. 25, 2020–Jan. 19, 2021), we have omitted the consideration of vaccination as the initial recorded vaccination data point in CDC’s COVID-19 vaccination data^28^ for Vermont is on Jan. 15, 2021.

**We did not consider vaccination (and thus vaccine effectiveness) for Period OS as the initial recorded data point in CDC’s COVID-19 vaccination data^28^ for Vermont is on Jan. 15, 2021. For Period D1 and Period D2, vaccine effectiveness is 88% and based on the effectiveness of two doses of the monovalent mRNA BNT162b2 (Pfizer-BioNTech, Comirnaty) against the Delta variant^15^.

^††^We assume immunity lasts 180 days on average^16^.

^‡‡^Represents a weighted sum of the base case’s median number of days from infection to isolation and the contacts’ median number of days from exposure to quarantine. See Supplementary Table S2 for more details.

Based on those user-defined inputs, users can estimate the combined effect of nonpharmaceutical interventions (NPIs; *e.g.*, facemask policies, social distancing) on transmission; then by setting CICT program performance to zero, simulate an epidemic curve in the absence of CICT. By comparing epidemic curves with and without the CICT program, we can obtain an estimate of CICT’s impact (see Technical Appendix in online Supplementary Information for more details and see^2–4,12^ for other applications).

### Sensitivity analyses

We investigated how changing input values for some key variables might alter the results and thus identify the most influential input variables. First, we estimated the change in CICT’s impact that may be attributable to the shorter latent period associated with the Delta variant by using the original strain’s 3-day latent period instead of the 2-day latent period believed associated with the Delta variant^7,8^. Second, we investigated how a higher initial value of the basic reproduction number *R*_*0*_ associated with the Delta variant may have impacted our CICT estimates by varying transmissibility ± 10%. Note that the model adjusts the effective reproduction number *R*_*t*_ value on a daily basis as the pool of susceptible individuals decreases. The *R*_*0*_ value adjusted in the sensitivity analyses is the starting value. Third, we investigated the impact of vaccination by varying the documented level of fully vaccinated individuals by ± 10%. Fourth, we varied public compliance with isolation and quarantine guidelines. For example, pandemic fatigue might have decreased public compliance with CICT programs in the later periods of analysis, but self-isolation and self-quarantine might still have occurred even for cases and contacts that were not interviewed or notified by public health officials. Finally, we varied the model’s input for the number of days from infection to isolation or quarantine from 6 to 9 days (see Supplementary Table S2 for more details of this parameter). This key CICT performance metric—which is defined as a weighted sum of the number of days between infection and isolation of base cases and the number of days between exposure and quarantine of their contacts—reported in Vermont’s CICT data, varies from 7 days for Period OS and Period D2, to 9 days for Period D1 (Table 1). Note that because our model is deterministic, there is no statistical uncertainty arising from stochastic factors.

## Results

### Comparing the original variant to the delta variant

**Figure 2:**
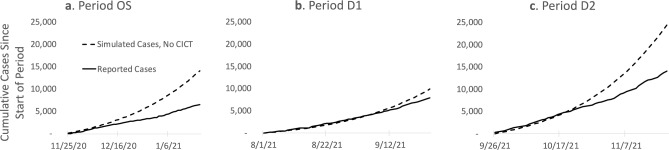
Comparison of case investigation and contact tracing’s (CICT) impact on the epidemic curves of various periods in Vermont, by dominating variant transmission, 2020–2021. The figure shows epidemic curves of the reported case count for the three study periods: Period OS, Period D1, and Period D2, with the case investigation and contact tracing (CICT) program in place and simulated epidemic curves to show what might have occurred had the CICT program not been implemented. The solid lines are Vermont’s reported cumulative case counts since the beginning of each study period with both CICT and other nonpharmaceutical interventions (NPIs) implemented. The dashed lines are the estimated curves illustrating the cumulative case count of what might have occurred in Vermont if CICT had not been implemented, and only other NPIs were implemented during the study period. The difference between any two lines within one panel represents the cases averted by CICT.

During the study period covering the original strain of the virus (Period OS: Nov. 25, 2020 to Jan. 29, 2021), we estimated that 7810 cases were averted by CICT which represents 55.4% of all COVID-19 cases that would have occurred in the absence of the CICT program (Table 2 and Fig. 2). During the two periods covering the Delta wave (Period D1: Aug. 1 to Sept. 25, 2021; and Period D2: Sept. 26 to Nov. 20, 2021), we estimated that 1437 and 9970 cases were averted by the CICT program for Period D1 and Period D2, respectively—these numbers represent 14.6% and 40.4% of all COVID-19 cases expected to occur in the absence of the CICT program (Table 2 and Fig. 2).

**Table 2:** Comparison of case investigation and contact tracing’s (CICT) impact during various periods in Vermont, by dominating variant transmission, 2020–2021.

Dominant strain	Original strain	Delta variant	Delta variant
Study period (Dates covered)	Period OS (Nov. 25, 2020–Jan. 19, 2021)﻿	Period D1 (Aug. 01–Sept. 25, 2021)﻿	Period D2 (Sept. 26–Nov. 20, 2021)﻿
**Estimated CICT impact**			
Cases averted	7810	1437	9970
Percentage reduction in COVID-19 cases*	55.4%	14.6%	40.4%

*The number of cases averted by CICT among every 100 cases not averted by vaccine or other nonpharmaceutical interventions (NPIs; such as facemask policies, large gathering restrictions, and school/business closures).

### The effect of latent period duration and basic reproduction number

We found that a shorter latent period of 2 days, compared to 3 days associated with the original variant, reduced the impact of CICT (Table S3) during the Delta periods. There was a 6.0 percentage point decrease in the number of cases averted during Period D1 (from 20.6% to 14.6%) and an 8.8 percentage point decrease during Period D2 (from 49.2% to 40.4%).

**Figure 3:**
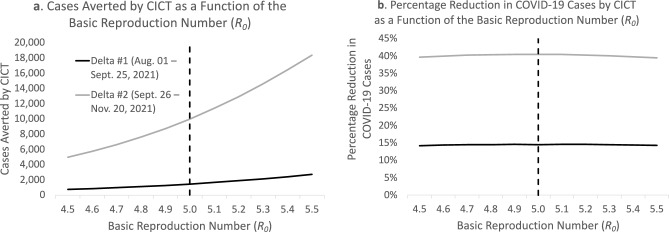
Impact of case investigation and contact tracing (CICT) during two Delta periods in Vermont, for different initial values of the basic reproduction number (*R*_*0*_). The results are presented in terms of Cumulative number of cases averted (Panel **A**) and corresponding percentage reduction in COVID-19 cases (Panel **B**). The results corresponding to the observed percentage of vaccinated population (Table 2) are represented by the dashed vertical line. Note that the model adjusts the *R*_*0*_ value on a daily basis as the pool of susceptible individuals decreases; the *R*_*0*_ value adjusted in the sensitivity analyses is the “starting” value. Further note the percentage reduction in COVID-19 cases (panel **B**) represents the number of cases averted by CICT among every 100 cases not averted by vaccine or other nonpharmaceutical interventions (NPIs; such as facemask policies, large gathering restrictions, and school/business closures).

We also investigated how much of the differences in our estimated CICT impact in Table 2 are attributable to changes in the initial value of the basic reproduction number, *R*_*0*_. We found that the higher transmissibility associated with the Delta variant^9,10^ increases the number of cases averted by CICT—this may be because each averted case prevents more new infections than with the original strain (Fig. 3, Panel A). However, because every case not reached by the CICT program now infects, on average, more individuals, the program’s relative impact (the percentage reduction in COVID-19 cases) is not affected in a meaningful way (Fig. 3, Panel B).

### The effect of differences in non-viral characteristics across periods

Vaccination reduces the pool of susceptible individuals that can benefit from CICT. Because mass vaccination in the U.S. started after our earliest period of analysis (Period OS), CICT’s impact was affected by vaccinated individuals only during Delta periods. Our sensitivity analysis of varying the documented level of fully vaccinated individuals by ± 10% shows, while the absolute number of cases averted by CICT is considerably affected by the size of the pool of susceptible individuals, the relative impact that CICT has on disease transmission is not affected in a meaningful way (Fig. S1). The assumed level of public compliance with isolation and quarantine guidelines (see Table S4 for baseline assumptions) also affected the results. For example, lowering the compliance among cases and contacts by 20 and 10 percentage points, respectively, resulted in over 25% reduction in the proportion of cases averted by CICT (Table S5).

The estimated reduction in expected COVID-19 cases by CICT during Period D1 was considerably lower than the reductions during Period OS and Period D2: 14.6% versus 55.4% and 40.4%, respectively (Table 2 and Fig. 2). We found that the main source of this divergence in CICT impact between Period D1 and Period D2 is the difference in the median number of days from infection to isolation/quarantine (Table S6; see Table S2 for more details on differences across periods and for a more detailed explanation of this key metric). During Period D1, the median number of days from infection to isolation/quarantine was nine days, two days longer than both Period OS and Period D2 (Table 2). Had Vermont’s CICT program succeeded in isolating/quarantining cases/contacts within seven days during the first Delta period (matching the speed in Period OS and Period D2), then percentage reductions in COVID-19 cases would be 36.6%, similar to the 40.4% achieved in the second Delta period (Table S6).

## Discussion

We estimated the effectiveness of Vermont’s CICT program to avert COVID-19 cases during periods dominated by two different SARS-CoV-2 variants. In the context of both the original SARS-CoV-2 strain and the Delta variant, CICT was successful as a public health tool in its ability to avert cases and mitigate transmission of disease. However, CICT had an overall greater effectiveness to avert cases during the period of original SARS-CoV-2 strain predominance. This conclusion aligns with two studies in Asia that demonstrated overall decreased effectiveness of CICT during outbreaks caused by the Delta variant^18,19^. Our findings suggest that the difference in CICT effectiveness during these periods is only partially explained by differences in the strains’ viral characteristics; while the shortened latency period of Delta variant did reduce CICT effectiveness 6–9 percentage points of COVID-19 cases averted by CICT, CICT’s ability to avert new cases was not meaningfully impacted by Delta variant’s higher *R*_*0*_. Non-viral factors, including the public’s adherence to isolation and quarantine guidelines and the speed of case and contact notification, contributed to the difference in CICT effectiveness as well. Even a one-day delay of case/contact notification, for example, was associated with up to a 15 percentage points decrease in COVID-19 cases averted by CICT. Overall, these findings suggest that while more transmissible variants do diminish the effectiveness of CICT, this public health tool still has the power to mitigate viral transmission—especially if certain performance metrics and the public’s adherence to isolation and quarantine recommendations are maintained.

Our study has limitations. First, we assumed, for each period studied, that the impact of CICT and other pharmaceutical (vaccination) and nonpharmaceutical interventions (*e.g.,* facemask policies) remained constant over the periods of study. To reduce the potential impact of such assumption, we limit our study periods to 56 days. Second, we do not account for partial immunity (*e.g.,* if an individual received their first vaccine shot during the study period), and hence assume individuals are either fully susceptible (because the individual was never vaccinated or never infected, or because immunity acquired through vaccination or prior infection was more than 180 days ago and is no longer protective) or fully immune (due to prior infection or vaccination). Third, we do not incorporate underreported cases in our model due to the absence of a reliable estimate for Vermont. While this might lead to an overestimation of CICT impact, the qualitative nature of our findings remains valid if there is no variation in the degree of underreporting across periods. Note that while we know polymerase chain reaction (PCR) test positivity—which proxies for underreported cases^20^—increased from 2.89% in Period OS, to 3.15% and 3.25% in Period D1 and Period D2, respectively (Table S1), we do not have precise data on underreporting. Therefore, assuming there is no variation in underreporting across periods is currently our most informed conjecture. Fourth, we model no differences in disease transmission that are attributable to age or location—in other words, we assume every individual in the population has the same risk of catching COVID-19 and behaves in the same way as any other individual. The model is deterministic, implying, for example, that the average number of infections caused by an infected individual is constant and consequently it overlooks stochastic elements such as super spreader events^21^ which may have a substantial role in a small population like Vermont’s. Fifth, our sensitivity analyses omit the consideration that rising disease burden may diminish CICT effectiveness due to limited CICT staff and time (see^22^ for the role of resource availability in outbreak control), though CICT capacity limit does not seem to have been an issue for most of the time covered in our analyses^23^. Sixth, the Vermont population modeled is assumed to be closed in the sense that there are no importations of infected individuals (*e.g.*, from a neighboring state). Additionally, differences and similarities in our estimates of CICT’s impact among the three periods cannot be solely attributed to the influence of variants’ characteristics. For example, differences in CICT’s performance might have been influenced by differences in unmeasured confounding factors such as program staff experience and efficiency, and capacity to handle the caseloads. Finally, this study was conducted using data from the U.S. state of Vermont, a jurisdiction that interviewed a higher percentage of cases and contacts than most other U.S. states^23,24^. Therefore, it is unclear how generalizable these results are to other jurisdictions. Interested parties, however, can use the publicly available tool and instructions available in Rainisch et al.^3^ to replicate this analysis for their own jurisdiction.

Our results indicate that isolation and quarantine remain effective tools for reducing the burden of COVID-19, despite more contagious and rapidly spreading emerging variants. Our work demonstrates the importance of isolation and quarantine to help mitigate the burden of COVID-19 in our communities. These results also illustrate the value of continuing to explore, even in non-pandemic times, ways to improve the impact of CICT activities.

### Supplementary Information

Supplementary Information.

## Data Availability

All data needed to evaluate the conclusions in the paper are present in the paper and Supplementary Materials.

## References

[1] Eames, K. T. & Keeling, M. J. Contact tracing and disease control. *Proc. Royal Soc. Lond. Series B Biol. Sci.* **270**(1533), 2565–2571 (2003). 10.1098/rspb.2003.2554 PMC1691540 14728778

[2] Jeon, S. *et al.* Estimates of cases and hospitalizations averted by COVID-19 case investigation and contact tracing in 14 health jurisdictions in the United States. *J. Public Health Manag. Pract.* **28**(1), 16–24 (2022). 34534993 10.1097/PHH.0000000000001420 PMC11334148

[3] Rainisch, G. *et al.* Estimated COVID-19 cases and hospitalizations averted by case investigation and contact tracing in the US. *JAMA Netw. Open* **5**(3), e224042–e224042 (2022). 35333362 10.1001/jamanetworkopen.2022.4042 PMC8956978

[4] Jeon, S. *et al.* Adapting COVID-19 contact tracing protocols to accommodate resource constraints, Philadelphia, Pennsylvania, USA, 2021. *Emerg. Infect. Dis.* **30**(2), 333 (2024). 38181801 10.3201/eid3002.230988 PMC10826771

[5] Kraemer, M. U. *et al.* Monitoring key epidemiological parameters of SARS-CoV-2 transmission. *Nat. Med.* **27**(11), 1854–1855 (2021). 34750555 10.1038/s41591-021-01545-w

[6] Bugalia, S., Tripathi, J. P. & Wang, H. Mutations make pandemics worse or better: Modeling SARS-CoV-2 variants and imperfect vaccination. *arXiv preprint *arXiv:2201.06285*,* (2022). 10.1007/s00285-024-02068-x 38507066

[7] Wang, Y. *et al.* Transmission, viral kinetics and clinical characteristics of the emergent SARS-CoV-2 Delta VOC in Guangzhou, China. *EClinicalMedicine* **40**, 101129 (2021). 34541481 10.1016/j.eclinm.2021.101129 PMC8435265

[8] Li, B. *et al.* Viral infection and transmission in a large, well-traced outbreak caused by the SARS-CoV-2 delta variant. *Nat. Commun.* **13**(1), 1–9 (2022). 35075154 10.1038/s41467-022-28089-y PMC8786931

[9] Liu, Y. & Rocklov, J. The reproductive number of the delta variant of SARS-CoV-2 is far higher compared to the ancestral SARS-CoV-2 virus. *J. Travel Med.* **28**(7), taab124 (2021). 34369565 10.1093/jtm/taab124 PMC8436367

[10] Alimohamadi, Y., Sepandi, M. & Esmaeilzadeh, F. Estimate of the basic reproduction number for Delta variant of SARS-CoV-2: A systematic review and meta-analysis. *J. Biostat. Epidemiol.* **8**(1), 1–7 (2022).

[11] U.S. Centers for Disease Control and Prevention, COVIDTracer and COVIDTracer advanced, 19 January 2021. [Online]. Available: https://www.cdc.gov/coronavirus/2019-ncov/php/contact-tracing/COVIDTracerTools.html. [Accessed 5 April 2023].

[12] Jeon, S. *et al.* Estimated cases averted by COVID-19 electronic exposure notification–Pennsylvania, November 8, 2020 to January 2, 2021. *Emerg. Infect. Dis.* **29**(2), 426 (2023). 36639132 10.3201/eid2902.220959 PMC9881797

[13] Castonguay, F. M. *et al. *Estimated public health impact of concurrent mask mandate and vaccinate-or-test requirement in Illinois, October to December 2021. *BMC Public Health ***24**(1), 1013 (2024). 38609903 10.1186/s12889-024-18203-8 PMC11010411

[14] CoVariants (c) 2020–2023 Emma Hodcroft, CoVariants: SARS-CoV-2 Mutations and Variants of Interest. [Online]. Available: https://covariants.org/per-country?region=United+States&country=Vermont. [Accessed 5 April 2023].

[15] Lopez Bernal, J. *et al.* Effectiveness of Covid-19 vaccines against the B. 1.617. 2 (Delta) variant. *N. Engl. J. Med.* **385**(7), 585–594 (2021). 34289274 10.1056/NEJMoa2108891 PMC8314739

[16] Levin, E. G. *et al.* Waning immune humoral response to BNT162b2 Covid-19 vaccine over 6 months. *N. Engl. J. Med.* **385**(24), e84 (2021). 34614326 10.1056/NEJMoa2114583 PMC8522797

[17] Rosenblum, H. G. *et al.* Interim recommendations from the advisory committee on immunization practices for the use of bivalent booster doses of COVID-19 vaccines—United States, October 2022. *MMWR Morb. Mortal. Wkly. Rep.* **71**(45), 1436–1441 (2022). 36355612 10.15585/mmwr.mm7145a2 PMC9707353

[18] Ma, X. *et al.* Contact tracing period and epidemiological characteristics of an outbreak of the SARS-CoV-2 Delta variant in Guangzhou. *Int. J. Infectious Dis.* **117**, 18–23 (2022). 35101637 10.1016/j.ijid.2022.01.034 PMC8800156

[19] Nguyen, T.-P. *et al.* Rapid impact assessments of COVID-19 control measures against the Delta variant and short-term projections of new confirmed cases in Vietnam. *J. Global Health* **11**, 03118 (2021). 10.7189/jogh.11.03118 PMC8693161 34987776

[20] Chiu, W. A. & Ndeffo-Mbah, M. L. Using test positivity and reported case rates to estimate state-level COVID-19 prevalence and seroprevalence in the United States. *PLoS Comput. Biol.* **17**(9), e1009374 (2021). 34491990 10.1371/journal.pcbi.1009374 PMC8448371

[21] Lewis, D. Superspreading drives the COVID pandemic–and could help to tame it. *Nature* **590**(7847), 544–547 (2021). 33623168 10.1038/d41586-021-00460-x

[22] Bugalia, S., Tripathi, J. P. & Wang, H. Mathematical modeling of intervention and low medical resource availability with delays: Applications to COVID-19 outbreaks in Spain and Italy. *Math. Biosci. Eng.* **18**(5), 5865–5920 (2021). 34517515 10.3934/mbe.2021295

[23] Borah, B. F. *et al.* High community transmission of SARS-CoV-2 associated with decreased contact tracing effectiveness for identifying persons at elevated risk of infection-vermont. *Clin. Infectious Dis.* **75**(Supplement_2), S334–S337 (2022). 10.1093/cid/ciac518 PMC9278248 35748711

[24] Lash, R. R. *et al.* COVID-19 case investigation and contact tracing in the US, 2020. *JAMA Netw. Open* **4**(6), e2115850–e2115850 (2021). 34081135 10.1001/jamanetworkopen.2021.15850 PMC8176334

[25] Xiang, Y. *et al.* COVID-19 epidemic prediction and the impact of public health interventions: A review of COVID-19 epidemic models. *Infectious Dis. Modell.* **6**, 324–342 (2021). 10.1016/j.idm.2021.01.001 PMC7790451 33437897

[26] Centers for Disease Control and Prevention (CDC). *COVID-19 Pandemic Planning Scenarios* (U.S Department of Health and Human Services, 2020).

[27] Liu, Y., Gayle, A. A., Wilder-Smith, A. & Rocklov, J. The reproductive number of COVID-19 is higher compared to SARS coronavirus. *J. Travel Med.* 1–4 (2020). 10.1093/jtm/taaa021 PMC7074654 32052846

[28] Centers for Disease Control and Prevention, COVID-19 Vaccinations in the United States, County, [Online]. Available: https://data.cdc.gov/Vaccinations/COVID-19-Vaccinations-in-the-United-States-County/8xkx-amqh. [Accessed 25 April 2023].

[29] Tahamtan, A. & Ardebili, A. Real-time RT-PCR in COVID-19 detection: Issues affecting the results. *Expert Rev. Mol. Diagn.* **20**(5), 453–454 (2020). 32297805 10.1080/14737159.2020.1757437 PMC7189409

[30] Bugalia, S., Tripathi, J. P. & Wang, H. Estimating the time-dependent effective reproduction number and vaccination rate for COVID-19 in the USA and India. *Math. Biosci. Eng* **20**(3), 4673–4689 (2023). 36896517 10.3934/mbe.2023216

[31] Smith, L. E., Potts, H. W., Amlot, R., Fear, N. T., Michie, S. & Rubin, G. J. Adherence to the test, trace, and isolate system in the UK: Results from 37 nationally representative surveys. *BMJ* **372**(608), (2021). 10.1136/bmj.n608 PMC8010268 33789843

[32] Park, C. L. *et al.* Americans’ COVID-19 stress, coping, and adherence to CDC guidelines. *J. General Intern. Med.* **35**(8), 2296–2303 (2020). 10.1007/s11606-020-05898-9 PMC7259430 32472486

[33] Pew Research Center, *The Challenges of Contact Tracing as U.S. Battles COVID-19*. Washington D.C., (2020).

[34] Castonguay, F. M. *et al.* Building a simple model to assess the impact of case investigation and contact tracing for sexually transmitted diseases: Lessons From COVID-19. *AJPM Focus* **3**(1), 100147 (2024). 38149077 10.1016/j.focus.2023.100147 PMC10749878

[35] Akman, O. *et al.* The hard lessons and shifting modeling trends of COVID-19 dynamics: Multiresolution modeling approach. *Bull. Math. Biol.* **84**, 1–30 (2022). 10.1007/s11538-021-00959-4 PMC8602007 34797415

[36] Chen, M. *et al.* The introduction of population migration to SEIAR for COVID-19 epidemic modeling with an efficient intervention strategy. *Inf. Fusion* **64**, 252–258 (2020). 32834796 10.1016/j.inffus.2020.08.002 PMC7406520

[37] Edridge, A. W. *et al.* Seasonal coronavirus protective immunity is short-lasting. *Nat. Med.* **26**(11), 1691–1693 (2020). 32929268 10.1038/s41591-020-1083-1

