# Supplementary material for: The public health impact of COVID-19 variants of concern on the effectiveness of contact tracing in Vermont, United States
Source: Sci Rep. 2024 Aug 1;14:17848. doi: 10.1038/s41598-024-68634-x (PMC11294356; doi:10.1038/s41598-024-68634-x)
Supplement: Supplementary file 1 — Supplementary Information. [file 41598_2024_68634_MOESM1_ESM.pdf]

Supplementary Information

The Public Health Impact of COVID-19 Variants of Concern on the Effectiveness of Contact Tracing in Vermont, United States

François M. Castonguay, PhD[1,2,3,*]; Brian F. Borah, MD[4,5]; Seonghye Jeon, PhD[1,2]; Gabriel Rainisch, PhD[1,2]; Patsy Kelso, PhD[4]; Bishwa B. Adhikari, PhD[1,2]; Daniel J. Daltry, PhD[4]; Leah S. Fischer, PhD[1,2]; Bradford Greening Jr., PhD[1,2]; Emily B. Kahn, PhD[1,2]; Gloria J. Kang, PhD[1,2]; Martin I. Meltzer, PhD[1,2]

[1]National Center for Emerging and Zoonotic Infectious Diseases, Division of Preparedness and Emerging Infections, Health Economics and Modeling Unit; Centers for Disease Control and Prevention, U.S. Department of Health and Human Services, Atlanta, GA.

[2]Modeling Support Team, Contact Tracing and Innovation Section (CTIS), State Local Tribal and Territorial (STLT) Task Force, CDC COVID-19 Response; Centers for Disease Control and Prevention, U.S. Department of Health and Human Services, Atlanta, GA.

[3]Department of Health Management, Evaluation and Policy, University of Montreal School of Public Health, and Centre for Public Health Research – CReSP; 7101 Av du Parc, 3e étage, Montréal, QC H3N 1X9, Canada.

[4]Vermont Department of Health.

[5]Epidemic Intelligence Service; Centers for Disease Control and Prevention, U.S. Department of Health and Human Services, Atlanta, GA.

[*]**Corresponding author:** francois.castonguay@umontreal.ca

Table S1. COVID-19 test positivity and share of polymerase chain reaction (PCR) tests in Vermont.

**Evaluation period (Dominant strain)**	**Period OS (Original strain)**	**Period D1 (Delta variant)**	**Period D2 (Delta variant)**
**Evaluation date**	11/25/20 – 1/19/21	8/1 – 9/25/21	9/26 – 11/20/21
**Test Positivity (%)**			
Total Lab Tests	3.07%	3.32%	3.36%
PCR Lab Tests	2.89%	3.15%	3.25%
**Share of PCR Lab Tests (%)**			
Total Lab Tests	91.50%	93.40%	94.57%
Positive Lab Tests	86.30%	88.68%	91.63%

Notes. Data were provided directly from the Vermont Department of Health.

Table S2. COVID-19 burden, observed CICT program metrics, and calculated CICT effectiveness in Vermont.

**Evaluation period (Dominant strain)**	**Period OS (Original strain)**	**Period D1 (Delta variant)**	**Period D2 (Delta variant)**
**Evaluation date**	11/25/20 – 1/19/21	8/1 – 9/25/21	9/26 – 11/20/21
**COVID-19 burden**			
Mean daily incidence*	19/100k pop	23/100k pop	40/100k pop
Reported cases	6,559	7,901	14,106
**% Population fully vaccinated**	<1%	66%	68%
**Observed performance metrics**			
**Community receptivity to CICT**			
% of cases interviewed	84%	83%	78%
% of interviewed cases naming contacts	90%	80%	84%
Timing of specimen collection (days post symptom onset)	2 days	2 days	2 days
**CICT program performance metrics**			
% of all contacts identified†	76%	67%	65%
% of identified contacts notified	91%	53%	52%
Timing of test results notification (days post specimen collection)‡	2 days	3 days	2 days
Timing of contact notification (days post specimen collection)§	2 days	4 days	2 days
**Calculated CICT effectiveness**			
% of cases and contacts isolated¶	43.0%	36.1%	33.9%
Days from infection to isolation or quarantine#	7 days	9 days	7 days

*Mean daily incidence for the 56 days starting from the beginning of the evaluation.

†% of contacts identified = # of named contacts / expected # of contacts per case.
Expected # of contacts per case = # of reported cases * average # named contacts per case

‡This is the reported median days from specimen collection to positive test results reported to health departments.

§This is the reported median days from specimen collection to contact notification.

¶Including contacts who later become cases. Calculated as follows using the observed performance metrics in this table, assumed compliance with isolation and quarantine guidance among cases and contacts in Table S4, and an assumed k=1.2:

[(% Cases interviewed * Compliance) + k ∗ % Contacts identified
* (% Contacts monitored * Compliance + % Contacts notified but not monitored * Compliance)] /(1+k)

where k is approximated from the effective reproduction number (R_e), since undetected infected contacts will infect R_e additional individuals on average. If the assumed compliance was 100%, the estimated effectiveness could be as high as 57% for Period D1, and 54% for Period D2.

#The average length of time from infection to isolation and quarantine between cases and contacts which later became cases. We assumed a 4 or 5-day pre-symptomatic period, depending on the latent period. We further assumed that interviewed cases and notified contacts to begin isolation and quarantine the day after their interactions with the health department. For more details, please refer to: Jeon *et al.* [2] , Technical Appendix, Figure S2.

Table S3. Comparison of CICT's Impact on COVID-19 cases for 2-day and 3-day latent duration, Vermont, 2020-2021.

	**2-day latent period (associated with the Delta variant) Baseline results***	**3-day latent period (Associated with the original strain) Sensitivity analysis**
**CICT impact estimate, Period D1**		
Cases averted	1,437	1,497
Percentage reduction in COVID-19 cases†	14.6%	20.6%
**CICT impact estimate, Period D2**		
Cases averted	9,970	10,931
Percentage reduction in COVID-19 cases†	40.4%	49.2%

*Results corresponding to a 2-day latent period associated with the Delta strain in the base case—see Table 2.
†The number of cases averted by CICT among every 100 cases not averted by vaccine or other nonpharmaceutical interventions (NPIs; such as facemask policies, large gathering restrictions, and school/business closures).

The Effect of Vaccination

Vaccination reduces the pool of susceptible individuals that can benefit from CICT. We expect CICT's impact to diminish when the number of vaccinated individuals increases as CICT programs may be unable to identify and prioritize unvaccinated contacts. Because there were few vaccinated individuals during our earliest period of analysis (Nov. 25, 2020 – Jan. 19, 2021), we expect CICT's impact to be affected by the presence of vaccinated individuals only during the two periods of analysis when the Delta strain dominated virus circulation (Aug. 01 – Sept. 25, 2021, and Sept. 26 – Nov. 20, 2021). To make sure we did not overestimate the impact of CICT during the two Delta periods, we removed the number of people vaccinated from the pool of susceptible individuals at the beginning of each analysis. In other words, to avoid an overestimation of CICT impact, we assumed that vaccine effectiveness was 100% in the pre-analysis period. Obviously, there are still breakthrough infections that are occurring, but this phenomenon is captured by our fitting process. Lastly, we assumed vaccine-induced immunity lasted for exactly 180 days, while in practice it fades gradually over time [15]. We assessed the influence on our results of uncertainty in Vermont's vaccination levels by varying the documented level of Vermont's fully vaccinated

population by ±10%. We found that the absolute number of cases averted is considerably affected by the size of the pool of susceptible individuals (Figure S1, Panel A), where susceptible individuals are defined as those who have never been infected or never vaccinated, and those whose disease- or vaccine-induced immunity has waned. While the absolute number of individuals is considerably affected by the size of the pool of susceptible individuals, the relative impact that it has on disease transmission is not affected in a meaningful way (Figure S1, Panel B).

Figure S1. Number of cases averted and corresponding percentage reduction in COVID-19 cases by case investigation and contact tracing (CICT) during two Delta periods in Vermont, for different levels of vaccination coverage.

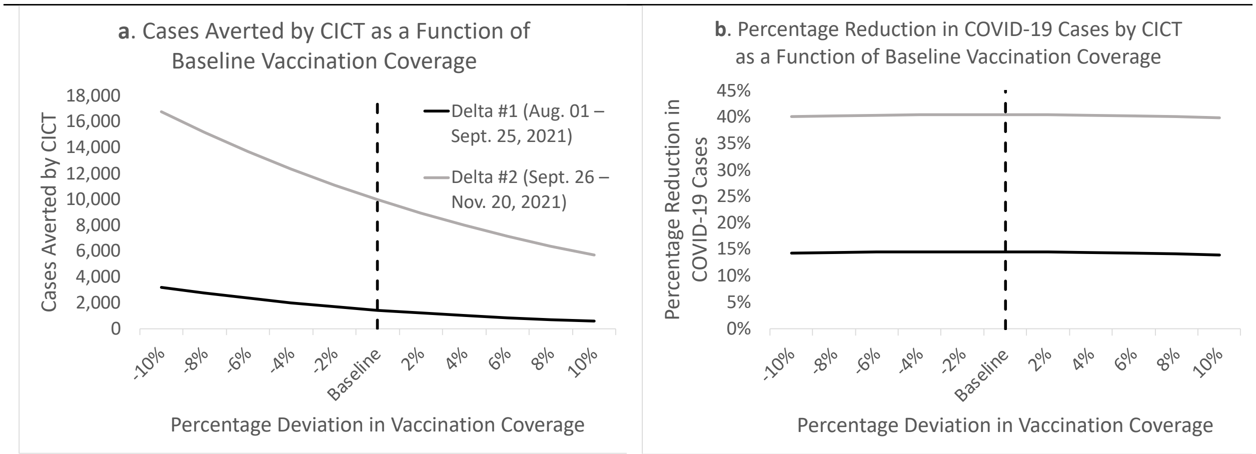

Notes. The results corresponding to the observed percentage of vaccinated population (Table 2) are represented by the dashed vertical line. The percentage reduction in COVID-19 cases (panel b) represents the number of cases averted by CICT among every 100 cases not averted by vaccine or other nonpharmaceutical interventions (NPIs; such as facemask policies, large gathering restrictions, and school/business closures).

The Effect of Public Compliance with Isolation and Quarantine

We assumed that 80% of cases who completed case interviews complied with the isolation guidelines and that 30% of contacts that are notified complied with quarantine guidelines (Table S4). We further assumed that confirmed cases who were not interviewed and contacts that were

not notified by the Vermont Department of Health did not isolate or quarantine. We varied these assumed values to see how this impacts the number of cases averted by the CICT program. First, we found that reducing the assumed percentage of cases who adhere to isolation guidelines by 20 percentage points (from 80% to 60%) and reducing the percentage of contacts who effectively quarantined by 10 percentage points (from 30% to 20%) decreased the percentage of cases averted from 14.5% to 10.8% for Period D1 (3.7 percentage point reduction) and from 40.1% to 31.0% for Period D2 (9.1 percentage point reduction); see Table S5. Second, we found a 20 percentage points increase in isolation for cases who did not complete case interviews (from 0% to 20%) and a 10 percentage points increase in quarantine of contacts who were not notified (from 0% to 10%), the percentage of cases averted increased from 14.5% to 15.7% for Period D1 (1.2 percentage point increase) and from 40.1% to 43.8% for Period D2 (3.7 percentage point increase); see Table S5. The lesser impact attributable to cases and contacts who were not in touch with the Vermont Department of Health is likely because these individuals represent a very low share of total cases and contacts due to the excellent public receptivity and CICT program effectiveness in Vermont (see Table S2).

Table S4. Assumed proportions of confirmed cases and their contacts that effectively isolated or quarantined*

**Confirmed cases that completed case interview**	80%
**Confirmed cases that did not complete case interview†‡**	0%
**Contacts that are notified**	30%
**Contacts that are not notified by their health department‡**	0%

Notes. Each row is a mutually exclusive group of cases or contacts. The sum of each row does not add up to 100%, as the numbers represent the assumed compliance within each group. 0% compliance means none of the cases or contacts in a group isolated or quarantined effectively. 100% means all cases or contacts in a group isolated or quarantined effectively after being interviewed or contacted.

*Based on a review of the literature. Findings and sources were as follows:

A review of multiple cross-sectional population surveys in the UK suggests that 40-45% of people who had COVID-like symptoms self-reported fully complying with isolation guidance during their infectious periods [30].

A survey in the U.S. found that 85% of respondents who had COVID-like symptoms or tested positive stayed home (according to CDC guidelines) except to get medical care [31].

And a third survey, also in the U.S., found that 93% of adults said they would definitely (73%) or probably (20%) quarantine themselves for at least 14 days if told to do so by a public health official because they had the coronavirus (*i.e.*, they were confirmed cases, not just exposed contacts) [32].

†Includes cases that were not reached and those that were reached but who did not agree to be interviewed.

‡Compliance was set to zero for these case/contact groups categories because any transmission reductions from quarantine and isolation are not attributable to direct interactions with the health department's CICT staff, and therefore outside of the scope of this analysis. Their inclusion here is to help distinguish between the various cases/contacts types.

Table S5. Comparison of CICT's impact during various periods in Vermont under different assumed public compliance with isolation and quarantine guidelines.

	**Baseline***	**Sensitivity 1**	**Sensitivity 2**
Interviewed cases that effectively isolated	80%	60%	80%
Cases that did not complete interviews that effectively isolated	0%	0%	20%
Notified contacts that effectively quarantined	30%	20%	30%
Contacts that were not notified that effectively quarantined	0%	0%	10%
**Isolation and quarantine impact estimate,† Period OS**			
Cases averted	7,810	4,759	8,228
Percentage reduction in COVID-19 cases‡	55.4%	43.1%	56.7%
**Isolation and quarantine impact estimate,† Period D1**			
Cases averted	1,437	1,031	1,585
Percentage reduction in COVID-19 cases‡	14.6%	10.9%	15.9%
**Isolation and quarantine impact estimate,† Period D2**			
Cases averted	9,970	6,658	11,623
Percentage reduction in COVID-19 cases‡	40.4%	31.3%	44.1%

*Results corresponding to the base case assumed levels of public compliance levels to isolation and quarantine guidelines—see Table 2.

†Because we account for the possibility that confirmed cases that did not complete case interviews might have effectively isolated, and similarly because we account for the possibility that contacts that are not notified might have effectively quarantined, we changed the wording from "CICT Impact Estimate" to "Isolation and Quarantine Impact Estimate."

‡Equivalent to the number of cases averted by CICT among every 100 cases not averted by vaccine or other nonpharmaceutical interventions (NPIs; such as facemask policies, large gathering restrictions, and school/business closures).

The Effect of Case Interview and Contact Notification Speed

Table S6. Comparison of CICT's Impact for various median number of days from infection to isolation.

Median days from infection to isolation	**9 days**	**8 days**	**7 days**	**6 days**
**CICT impact estimate, Period D1**				
Cases averted	1,437*	2,351	3,604	5,089
Percentage reduction in COVID-19 cases†	14.6%*	23.9%	36.6%	51.7%
**CICT impact estimate, Period D2**				
Cases averted	4,465	6,868	9,970*	13,473
Percentage reduction in COVID-19 cases†	18.1%	27.9%	40.4%*	54.6%

*Results corresponding to the observed median days from infection to isolation during this period—see Table 2.
†Equivalent to the number of cases averted by CICT among every 100 cases not averted by vaccine or other nonpharmaceutical interventions (NPIs; such as facemask policies, large gathering restrictions, and school/business closures).

Technical Appendix

We use the U.S. Centers for Disease Control and Prevention (CDC)'s COVIDTracer Advanced modeling tool to estimate the public health impact of CICT in Vermont; see [2, 3, 4, 12, 33] for other papers that use or discuss this modeling tool. Using Vermont-specific data, the model simulates counterfactual epidemic curves without the CICT program (see Section on Modeling Nonpharmaceutical Interventions for more details). The difference between the observed case count and the model-generated case count without the CICT program, provides our estimate of the impact of CICT in terms of the number of cases averted (see Figure 2). We assume that transmission reductions due to nonpharmaceutical interventions (NPIs)—including CICT and other NPIs such as facemask policies, large gathering restrictions, school/business closures, etc.—remain constant over the period of analysis (56 days). The disease dynamics of COVID-19 are modeled using an SIR epidemiological model (see [24] for a detailed discussion of compartments models for COVID-19, and see [34] for more details on how such models can be used to model

contact tracing interventions and virus mutations), which simulates the change in the number of susceptible (S), infected (I), and removed (R) individuals over time.

Model of Disease Transmission

We use a susceptible–infected–removed (SIR) model to predict the disease dynamics of a jurisdiction (here, Vermont). We assume the jurisdiction's total population is closed in the sense that there are no exogeneous importations of infected individuals (*e.g.,* from a neighboring state; see [35] for instance) and that the jurisdiction's population size remains constant over the period of analysis (*i.e.*, we omit from births of susceptible individuals and deaths given the short time frame). Given the short time-horizon of our analyses (56 days), we assume immunity through vaccination (whether it is partial or full) remains at the same level over the period analyzed. This assumption simplifies the model-fitting process, as we do not have to account for complex vaccination dynamics.

In the absence of NPIs, the change in susceptible individuals between any two days is

$$\dot{S} = -\sum_{i=1}^{n} \beta_i S \frac{I_i}{N}$$

where β_i is the effective contact rate of individuals that were infected i days ago (n is the duration of infection, in days), S is the number of susceptible individuals, I_i is the number of infected individuals that were infected i days ago, N is the total size of the jurisdiction's population. An implication of the above equation is that we assume homogeneous mixing among individuals, which means we do not account for age- or location-based heterogeneities in transmission.

After being infected, individuals transition into the infected class I_1, where the change in individuals infected $i = 1$ day ago is

$$\dot{I}_1 = \sum_{i=1}^{n} \beta_i S \frac{I_i}{N} - \gamma I_1$$

and the change in individuals infected $i = 2, \dots, n$ days ago is

$$\dot{I}_i = \gamma I_{i-1} - \gamma I_i$$

where γ is the duration an individual spends in each infectious compartment (*i.e.*, one day). Note that we implicitly account for an exposed (E) class in the above infectious compartments by setting $\beta_i = 0$ for $i = 1, \dots, \ell$, where $\ell < n$ is the length (in days) of the latent period.

At the beginning of the analysis, the jurisdiction's fully protected population (*i.e.*, the "removed" individuals) is defined as the sum of (i) the individuals that were fully vaccinated within six months of the start date of the period of analysis and (ii) the individuals that were infected within the last six months (regardless of whether they were vaccinated). There are a few implicit assumptions that are embedded in this calculation. First, we assumed that both naturally acquired and vaccine-acquired immunity last for 180 days [36] and provide the same level of immunity. Second, the risk of getting infected is the same for individuals that were never vaccinated and for individuals who were vaccinated more than six months ago. Third, the likelihood of getting vaccinated is the same regardless of whether an individual was previously infected or not. Fourth, there is no partial immunity (*i.e.*, individuals are either fully protected or fully susceptible), and previously infected and vaccinated individuals cannot revert to being susceptible during the analytic periods analyzed (*i.e.*, the effects of waning immunity are insignificant over the 56 days of our study period). As a result, the number of removed individuals in the jurisdiction's population changes according to

$$\dot{R} = \gamma I_n$$

since immunity through vaccination is assumed constant over the period analyzed.

Modeling Nonpharmaceutical Interventions

The above model of disease transmission can be modified to account for transmission reductions due to CICT and other NPIs (see Figure S2 for a schematic representation of the disease transmission model with CICT). While non-CICT NPIs reduce by a certain proportion each of the contact rates β_i, CICT makes certain β_i's equal to zero with isolation or quarantine of infected individuals. Using the above notation, the basic reproduction number, R_0, of this model is

$$R_0 = \frac{\sum_{i=1}^{n} \beta_i}{\gamma}.$$

The model can disentangle the public health impact of the CICT program from the public health impact of all other NPIs. Transmission reductions from the CICT program (*i.e.*, CICT effectiveness) were calculated by inputting in the model two key jurisdiction-specific "performance values" which are: (i) the proportion of cases and contacts that entered isolation and quarantine, and (ii) the days required to do so (see Table S2). By assuming certain levels of compliance with isolation and quarantine guidelines, we can use the SIR model described above to obtain an estimate of the number of COVID-19 cases averted by CICT by comparing the scenarios with and without the CICT program. That is, in the case where there is no CICT program, we assume that the only cases and contacts who entered isolation and quarantine are those who did so voluntarily, and we compare the cumulative number of cases between the two scenarios. This way of calculating the public health impact of the CICT program implicitly implies that some cases and contacts would only go into isolation or quarantine if they had been interviewed or notified by the CICT program, and that the effect of the CICT program on the reduction in disease transmission is constant over the entire study period (56 days).

In addition to the transmission reduction due to the CICT program, there are also other, non-CICT, NPIs that might affect COVID-19 transmission (*e.g.*, school and business closures, large gathering restrictions). We estimate their impact on transmission reduction by fitting the curve of cumulative cases modeled with the above epidemiological model to the jurisdiction's reported cumulative cases. Essentially, what we are doing here is adding a value between 0 and 1 that multiplies each of the contact rates β_i. The value that minimized the deviation (*i.e.*, the transmission reduction that minimized the mean squared error) between the fitted and reported cumulative case curves is the estimate of the effectiveness of other NPIs. This fitting process gives us an estimated percentage reduction in transmission attributable to other NPIs. An implication of this fitting process is that the effects of other NPIs are implicitly constant over the entire study period (56 days).

Figure S2. COVIDTracer Advanced's Model Structure that distinguishes between cases and contacts that are traced or not traced, and symptomatic or asymptomatic.

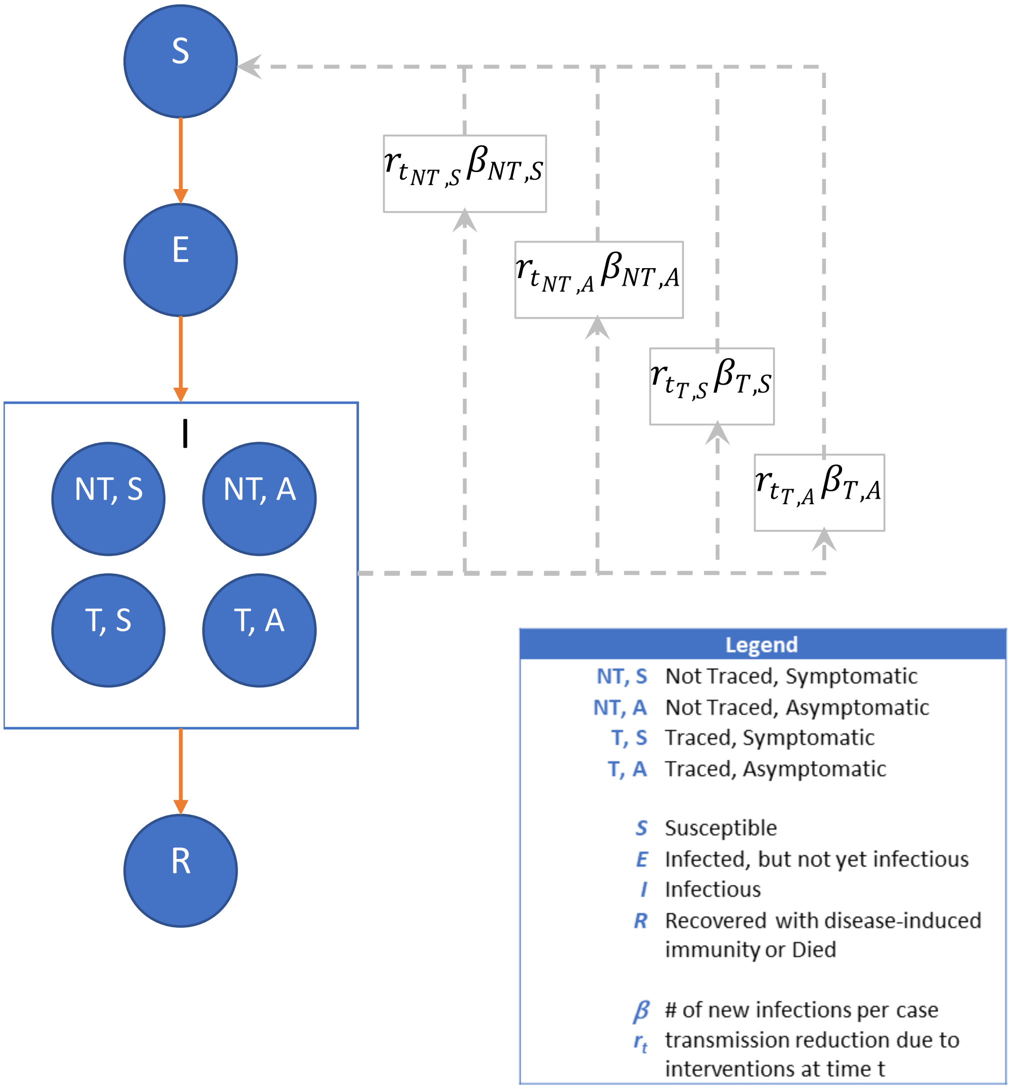

Notes: The model consists of individuals who are either *Susceptible* (S), *Infected but not yet Infectious* (E), *Infectious* (I), *Recovered* (R). Individuals can move between these compartments as indicated by the orange arrows. The rate of new infections is influenced by the number of individuals in the *Infectious* (I) category (depicted by the light grey dashed lines). There are 4 types of *Infectious* individuals: cases (symptomatic or asymptomatic) who adhere to isolation guidelines because they were engaged by their health departments via case investigation and contact tracing efforts (CICT), and cases (symptomatic or asymptomatic) who do not participate in CICT efforts. The overall risk to the *Susceptible* population of onward transmission is dependent upon both the distribution of cases among these 4 infectious categories on each day, and any reductions in transmission associated with a jurisdiction's implementation of CICT, and vaccine and other, non-pharmaceutical interventions.
